# A Comparison of Medical and Psychobehavioral Emergency Department Visits Made by Adults with Intellectual Disabilities

**DOI:** 10.1155/2012/427407

**Published:** 2012-09-29

**Authors:** Yona Lunsky, Rob Balogh, Alin Khodaverdian, Deborah Elliott, Christine Jaskulski, Susan Morris

**Affiliations:** ^1^Centre for Addiction and Mental Health, 501 Queen Street West, Toronto, ON, Canada M5V 2B4; ^2^University of Toronto, 27 King's College Circle, Toronto, ON, Canada M5S 1A1; ^3^Faculty of Health Sciences, University of Ontario Institute of Technology, 2000 Simcoe Street North, Oshawa, ON, Canada L1H 7L7; ^4^Centre for Addiction and Mental Health, 1001 Queen Street West, Toronto, ON, Canada M6J 1H4; ^5^Queen's University, Kingston, ON, Canada K7L 3N6

## Abstract

*Study Objective*. We describe and contrast medical to psychobehavioral emergency visits made by a cohort of adults with intellectual disabilities. *Methods*. This was a study of 221 patients with intellectual disabilities who visited the emergency department because of a psychobehavioral or medical emergency. Patient profiles are described and logistic regression was used to assess predictors of psychobehavioral emergencies in this group, including age, residence, psychiatric diagnosis, cognitive level, and life events. *Results*. Ninety-eight individuals had medical emergencies and 123 individuals presented with psychobehavioral emergencies. The most common medical issue was injury and the most common psychobehavioral issue was aggression. In the multivariate analysis, life events (odds ratio (OR) 0.28; 95% confidence interval (CI) 0.10 to 0.75), psychiatric diagnosis (OR 2.35; 95% CI 1.12 to 4.95), and age group (OR 4.97; 95% CI 1.28 to 19.38) were associated with psychobehavioral emergencies. Psychobehavioral emergencies were more likely to result in admission and caregivers reported lower rates of satisfaction with these visits. *Conclusion*. Emergency departments would benefit from greater understanding of the different types of presentations made by adults with intellectual disabilities, given the unique presentations and outcomes associated with them.

## 1. Introduction 

Compared to the general population, adults with intellectual disabilities experience poorer health and more difficulty in finding and receiving appropriate health care [1]. Perhaps the most challenging setting to serve these patients in is the emergency department, where, access to patient history is difficult, the time to develop relationships between patients and doctors is limited, and patients and caregivers are unprepared and in a state of crisis. Recent research [2, 3] and opinion papers [4] highlight that emergency department staff feel ill trained to manage these patients and that routine assessments and examinations can be complex due to patients' poor communication and functional limitations. 

Little is known about the extent to which individuals with intellectual disabilities access emergency departments although three recent studies have reported that such visits are more common in those with intellectual disabilities than the general population [5–7]. In a study of 186 emergency department users with intellectual disabilities, Lin et al. [6] found that the most common reasons for these visits were fever (27%), diarrhea (14.9%), injury (14.4%), seizure (14.4%), and asthma (7.5%). Venkat et al. [7] described a cohort of 431 individuals with intellectual disabilities living in congregate care settings, of which 222 visited the emergency department over 18 months. Compared to the general population, these individuals were more likely to visit due to digestive disorders or ill-defined symptoms. Interestingly, neither of these studies described individuals with psychiatric presentations.

Recent research has demonstrated that adults with both intellectual disabilities and psychiatric disorder are more likely to visit emergency departments than those with only one condition [5]. Psychobehavioral emergencies in those with intellectual disabilities are even more complex for physicians to assess than medical emergencies [8]. Triggers for such visits may be different than those for medical concerns, and patient characteristics may also differ. Qualitative research has suggested that aggression is the primary reason for emergency psychiatric visits in individuals with intellectual disabilities [3]. These visits are traumatic for both caregivers [9] and patients [10], and they can be triggered by clinical issues as well as life circumstances. To date, no quantitative studies have described such emergency visits by individuals with intellectual disabilities in contrast to medical visits.

The purpose of this study was twofold: first, to describe the medical and psychobehavioral presentations of a cohort of adults with intellectual disabilities who visited the emergency department, and secondly, to compare those individuals experiencing medical emergencies with those whose emergencies were predominately psychobehavioral in nature. The two groups were compared in terms of demographics, presenting concerns, outcome, and caregiver satisfaction. 

## 2. Materials and Methods

### 2.1. Study Group

This study was conducted in Ontario, Canada. In Canada, each province administers a tax-based health insurance plan that provides universal and comprehensive coverage for medically necessary hospital, emergency department, and physician and surgical-dental services. Ontario, in addition to having publicly funded healthcare, also funds community-based social services for people with intellectual disabilities. Specialized clinical services for intellectual disabilities, however, are limited. This study used data from 221 subjects who made an emergency department visit over a two-year period (June 2007–May 2009). The subjects were from a larger study of 750 adults with intellectual disabilities living in or close to urban centers in Ontario, Canada who had experienced at least one crisis during the study period, as reported by staff from thirty-four community agencies that provide mental health or social services to people with intellectual disabilities. All individuals whose crisis resulted in a visit to the hospital emergency department (*n* = 234) were eligible for inclusion in this study. Given that the purpose of this study was to compare those with psychobehavioral emergencies (123 individuals) to those with medical emergencies (98 individuals), we excluded 13 individuals from the original 234 whose crisis was categorized as both medical and psychobehavioral. If the individual had more than one emergency visit, the first visit was selected for analysis.

### 2.2. Instruments and Procedure

Staff from participating mental health and social service agencies, trained by the research team completed 2 forms. (1) *Client Background Form* includes information on patient demographics (age, gender, residential setting), medical and psychiatric diagnoses, and significant life events over the past year, based on a blank item life events list as part of the PAS ADD checklist [11] and (2) *Emergency Visit Form* includes items describing the emergency and the visit outcome (whether the patient was admitted or discharged) along with caregiver satisfaction with visit. Caregiver satisfaction with treatment given in the emergency department was recorded using a 5-point scale with 5 being most satisfied.

Staff were instructed to complete forms as soon as possible following the crisis and subsequently forward the forms, with no identifying information, to the research team. Standardized forms allowed for a greater level of detail on the patient background and the crisis itself than what is contained in hospital documentation. The emergency descriptions were reviewed by two raters and coded as medical only or psychobehavioral. All medical events were categorized according to ICD 9 general categories by two raters (AK, DE), as was done in a previous study by Venkat et al. [7] Psychobehavioral events were reviewed and categorized into one of 11 categories by the same raters (AK, DE): physical aggression/injury to others, suicidal behaviour/injury to self, suicidal ideation only, other psychiatric symptoms (e.g., panic or paranoia), verbal aggression only, property damage only, victim of verbal/physical abuse, missing/AWOL, sexual deviance, arson, respite/lack of resources, and other. When there was a discrepancy between raters, the case was discussed with a third rater (YL), and a consensus was reached. For both medical and psychobehavioral crisis categories, all categories with 5 or fewer individuals were aggregated into “other” category.

#### 2.2.1. Primary Data Analysis

We calculated descriptive statistics including proportions, means, standard deviations, and medians where appropriate. Logistic regression was used to assess the association between the dependent variable (psychobehavioral versus medical crisis) and independent variables. Both unadjusted and adjusted odds ratios are provided along with 95% confidence intervals. Chi square analysis was conducted to compare disposition between the two groups and caregiver satisfaction ratings were compared using a *t*-test. Statistical analyses were completed using SPSS version 15.0. 

## 3. Results

### 3.1. Characteristics of Study Subjects

221 individuals were reported to have visited the emergency department due to medical (*n* = 98) or psychobehavioral (*n* = 123) crisis. As illustrated in Table 1, patients presented with a variety of medical issues, most commonly injury. The most common psychobehavioral presentation was physical aggression, followed by suicidality. 

## 4. Main Results

Table 2 shows the unadjusted odds ratios for emergency department visits comparing psychobehavioral presentations to those medical in nature.  Table 3 shows the results of the multiple variable logistic regression using the same variables as in Table 2. Three comparisons remained significant in this analysis. Compared to persons greater than 45 years of age, persons less than 26 years of age had 5 times the odds of experiencing a psychobehavioral emergency (95%  CI = 1.3, 19.4). Having a psychiatric diagnosis (versus no psychiatric diagnosis) was associated with 2.4 increased odds of having a psychobehavioral emergency event. Lastly, having two or more life events (versus no life events) was a strong predictor of experiencing a psychobehavioral emergency (OR = 3.6, 95%  CI = 1.3, 10 (note: the reciprocal of the table result is used to simplify interpretation)). Closer investigation about the nature of life events revealed that the only type of life event in the prior year more frequent for those with medical emergencies was injury or illness (33% versus 9.8%; *χ* ^2^(1) 18.30, *P* < 0.001). All other life events were more common in those whose emergencies were psychobehavioral in nature.

Outcome of emergency department visit was examined in two ways: disposition and satisfaction. The proportion of individuals admitted to hospital from the emergency department visit differed, with individuals experiencing psychobehavioral emergencies having a greater likelihood of hospital admission (47.1% versus 26.0%; *χ* ^2^(1) 11.82, *P* = .001). Caregivers were less satisfied with outcome of the emergency visit for clients with psychobehavioral emergencies (*M* = 0.96, SD = 1.68) compared to medical emergencies (*M* = 2.46, SD = 1.87) (*t*(191) = 5.80, *P* < 0.001).

## 5. Discussion

This is the first study to describe and contrast emergency department visits for medical reasons with visits for psychobehavioral reasons by individuals with intellectual disabilities. Indeed, there are important differences between the people who present with each of these types of emergencies, including differences in visit outcome and satisfaction. When demographic and clinical variables were taken into account in a statistical model, significant predictors of psychobehavioral versus medical emergencies were age, psychiatric comorbidity, and previous life events. While admission rates were higher in the psychobehavioral emergency group, satisfaction rates were lower. This study illustrates, in contrast to previous studies with an exclusive focus on medical emergencies, that both types of visits occur for people with intellectual disabilities. Emergency departments, therefore, need to be prepared to respond to either type of presentation. 

There were some interesting findings when reviewing the types of emergencies most commonly experienced by this population. Our study found, in contrast to the Venkat study [7], that emergencies related to injuries were most common. This raises the question as to whether individuals with intellectual disabilities who have accidents are safe and receiving the level of support they require. Similar issues were raised in a recent Australian study on accidents requiring emergency response in youth with intellectual disabilities [12]. Further research on accidents/injuries in the adult population is warranted. With regard to psychobehavioral emergencies, aggression toward others was the most common type of presentation. Aggression may have an underlying psychiatric cause but can also be a way to communicate pain or discomfort, when language is limited, making comprehensive medical screening crucial [8]. Management of aggression is difficult in the emergency environment, and it would be important for staff to consider how to tailor the hospital environment for these situations [4], given their relative frequency. Use of restraint for these individuals can be very traumatizing [10] and it is therefore important to consider when and how restraints are used. 

It was not surprising that age and having a psychiatric diagnosis predicted psychobehavioral visits. As with the general population, older adults with intellectual disabilities have more medical problems and problems like aggression tend to decrease with age [13]. It is interesting that life events also predicted psychobehavioral visits. Life events have also been reported as predictors of psychiatric hospitalization in this population [14]. It is important for emergency physicians to recognize the potential role of life events in patients with intellectual disabilities and screen for them in the emergency assessment, particularly when the presentation does not appear to be medical in nature [8].

Serving patients with intellectual disabilities in the emergency department is complex. This paper suggests that from a caregiver perspective, the experience is more distressing and less satisfying when the trigger for the visit is psychobehavioral versus medical. This may be because medical concerns are more obvious to the emergency staff and more straightforward to diagnose and treat. Emotional or psychobehavioral issues are more challenging to assess, making the wait time for assessment very difficult, and the emergency department is not the ideal location to resolve them. Often what is needed are community resources, which hospital staff cannot easily access. It is worth examining in future research whether patients with intellectual disabilities and psychobehavioral presentations are more likely to return to hospital within a short period, given the complexity of their issues. 

This study has several limitations. All information reported here was gathered by community-based staff, whose perspectives on what occurs in hospital may differ from the opinions of hospital staff. Study authors rated emergencies based on the description, without knowing the final diagnosis given by the hospital staff. It would be important to supplement information in this study with chart audit information or data collected through direct observation of emergency assessments. This study focused on a single emergency visit per individual. Given that a significant proportion of individuals with intellectual disabilities have repeat visits to hospital, particularly those with psychiatric issues [5, 15], research is needed on patterns of visits over time. Finally, the small sample size in the study leads to large confidence intervals. It would be important to examine predictors of psychobehavioral emergencies in a larger population. It would also be important to examine these issues in other jurisdictions where health and social services to people with intellectual disability are delivered differently.

## 6. Conclusion

This study has important clinical and administrative implications. Although most of the literature on emergencies and intellectual disabilities focuses on medical issues, psychobehavioral emergencies also occur and can be quite complex. These emergencies tend to happen in younger individuals and can be preceded by life events. Emergency staff need adequate training on how to best respond to such emergencies. Guidelines and tools have been developed to assist in this process [8, 16] and it is important to consider how to foster their implementation in emergency departments. In addition to developing hospital-based strategies, alternatives to hospital visits for the management of psychobehavioral emergencies should also be explored. 

## Figures and Tables

**Table 1:** Proportion of visits by medical or psychobehavioral presentation category.

Medical crisis category	*N* = 98	Case example(s)
Injury	41 (41.8%)	Client ran across street on yellow light and was hit by a car
Ill-defined symptoms/signs	11 (11.2%)	Client became lethargic, was not eating, and was acting very much out of character
Nervous system/sense organs	10 (10.2%)	Client had 6 seizures in one day
Digestive	9 (9.2%)	Client was experiencing chronic constipation
Infectious/parasitic	7 (7.1%)	Client was vomiting all day and had a very high fever
Other (includes endocrine/nutritional/metabolic, musculoskeletal, circulatory, respiratory, genitourinary, pregnancy, and skin)	20 (20.4%)	Client needed to be assessed for a urinary tract infection Client broke out with skin irritation

Psychobehavioral crisis category	*N* = 123	Case example(s)

Physical aggression	40 (32.5%)	Son pushed mother against wall and hit her in the head
Suicidal ideation/behaviour	25 (20.3%)	Client argued with parent and then overdosed on psychotropic medications and was admitted to hospital
Other psychiatric symptoms	16 (13.0%)	Anxious, depressed, and experiencing hallucinations
Verbal aggression	9 (7.3%)	Client has been very verbally abusive these past few weeks. He had threatened to hurt roommate
Property damage	6 (4.9)	Client agitated, was angry, and trashed apartment
Other (e.g., missing, sexual deviance, and arson),	24 (19.5%)	Client was upset with a staff and eloped from her day program.

**Table 2:** Unadjusted odds ratios of association between variables and type of emergency visits (psychobehavioral versus medical crisis).

Variables	Total	Medical visit *N* (%)	Psychobehavioral visit *N* (%)	OR	95% CI
Age in years					
≤25	57 (25.8%)	11 (11.2%)	46 (37.3%)	8.13***	3.45–19.23
26–45	106 (48.0%)	48 (49.0%)	58 (47.2%)	2.35*	1.20–4.61
46+ (ref)	56 (25.3%)	37 (37.8%)	19 (15.4%)		
Sex					
Female	86 (38.9%)	39 (39.8%)	47 (38.2%)	0.94	0.54–1.61
Male (ref)	135 (61%)	59 (60.2%)	76 (61.8%)		
Level of disability					
Borderline/mild	88 (39.8%)	26 (35.1%)	62 (59%)	2.66**	1.44–4.93
Moderate/severe (ref)	91 (41%)	48 (64.9%)	43 (41%)		
Cultural background					
Caucasian	165 (74.6%)	79 (82.3%)	86 (71.7%)	0.27*	0.09–0.85
Other	31 (14.0%)	13 (13.5%)	18 (15%)	0.35	0.09–1.28
African Canadian (ref)	20 (9.0%)	4 (4.2%)	16 (13.3%)		
Residence					
Group home	91 (41.1%)	56 (57.7%)	35 (29.2%)	0.41*	0.22–0.77
Family	53 (23.9%)	12 (12.4%)	41 (34.2%)	2.25*	1.02–4.99
Minimal supports (ref)	73 (33.0%)	29 (29.9%)	44 (36.7%)		
Psychiatric diagnosis					
Yes	114 (51.5%)	33 (34%)	81 (65.9%)	3.75***	2.13–6.58
No (Ref)	106 (47.9%)	64 (66%)	42 (34.1%)		
Autism diagnosis					
Yes	53 (23.9%)	18 (18.6%)	35 (28.5%)	1.75	0.92–3.32
No (ref)	167 (75.5%)	79 (81.4%)	88 (71.5%)		
Life events					
0	47 (21.2%)	32 (33%)	15 (12.2%)	0.28*	0.14–0.58
1	56 (25.3%)	21 (21.6%)	35 (28.5%)	1.01	0.52–1.94
2 or more (ref)	117 (52.9%)	44 (45.4%)	73 (59.3%)		

*P < 0.05, **P < 0.01, ***P < 0.001; note: ref: reference category.

**Table 3:** Adjusted odds ratios of association between variables and type of emergency visits (psychobehavioral versus medical crisis).

Variables	OR	95% CI
Age in years		
≤25	4.97*	1.28–19.38
26–45	1.67	0.68–4.09
46+ (ref)		
Sex		
Female	1.17	0.53–2.57
Male (ref)		
Level of disability		
Borderline/mild	1.68	0.76–3.72
Moderate/severe (ref)		
Cultural background		
Caucasian	0.42	0.07–2.65
Other	0.57	0.08–4.25
African Canadian (ref)		
Residence		
Group home	0.65	0.25–1.64
Family	1.92	0.58–6.34
Minimal supports (ref)		
Psychiatric diagnosis		
Yes	2.35*	1.12–4.95
No (Ref)		
Autism diagnosis		
Yes	1.67	0.65–4.29
No (ref)		
Life events		
0	0.28*	0.10–0.75
1	1.09	0.43–2.73
2 or more (ref)		

*P < 0.05; note: ref: reference category.

## References

[1] United States Public Health Service, O. o. t. S. G. (2001). Closing the gap: a national blueprint to improve the health of persons with mental retardation. *Report of the Surgeon General's Conference on Health Disparities and Mental Retardation*.

[2] Iacono T, Davis R (2003). The experiences of people with developmental disability in Emergency Departments and hospital wards. *Research in Developmental Disabilities*.

[3] Lunsky Y, Gracey C, Gelfand S (2008). Emergency psychiatric services for individuals with intellectual disabilities: perspectives of hospital staff. *Intellectual and Developmental Disabilities*.

[4] Grossman SA, Richards CF, Anglin D, Hutson HR (2000). Caring for the patient with mental retardation in the emergency department. *Annals of Emergency Medicine*.

[5] Lunsky Y, Lin E, Balogh R (2011). Are adults with developmental disabilities more likely to visit EDs?. *American Journal of Emergency Medicine*.

[6] Lin JD, Yen CF, Loh CH (2006). A cross-sectional study of the characteristics and determinants of emergency care utilization among people with intellectual disabilities in Taiwan. *Research in Developmental Disabilities*.

[7] Venkat A, Pastin RB, Hegde GG, Shea JM, Cook JT, Culig C (2011). An analysis of ED utilization by adults with intellectual disability. *American Journal of Emergency Medicine*.

[8] Bradley E, Lofchy J (2005). Learning disability in the accident and emergency department. *Advances in Psychiatric Treatment*.

[9] Weiss JA, Lunsky Y, Gracey C, Canrinus M, Morris S (2009). Emergency psychiatric services for individuals with intellectual disabilities: caregivers’ perspectives. *Journal of Applied Research in Intellectual Disabilities*.

[10] Lunsky Y, Gracey C (2009). The reported experience of four women with intellectual disabilities receiving emergency psychiatric services in Canada: a qualitative study. *Journal of Intellectual Disabilities*.

[11] Moss S (2012). *PAS-ADD Checklist*.

[12] Sherrard J, Tonge BJ, Ozanne-Smith J (2001). Injury in young people with intellectual disability: descriptive epidemiology. *Injury Prevention*.

[13] Tyrell J, Dodd P, Davidson P (2003). Psychopathology in older age. *Mental Health, Intellectual Disabilities and the Aging Process*.

[14] Stack LS, Haldipur CV, Thompson M (1987). Stressful life events and psychiatric hospitalization of mentally retarded patients. *American Journal of Psychiatry*.

[15] Pasic J, Russo J, Roy-Byrne P (2005). High utilizers of psychiatric emergency services. *Psychiatric Services*.

[16] Sullivan W, Berg JM, Bradley E (2011). Primary care of adults with developmental disabilities: Canadian consensus guidelines. *Canadian Family Physician*.

